# Smart nanobiocatalysts for waste-to-biofuel conversion: integrating Nano–Bio interfaces and AI-driven design

**DOI:** 10.3389/fbioe.2026.1847169

**Published:** 2026-07-01

**Authors:** Hotaf Hassan Makki

**Affiliations:** Biology Department, Faculty of Science, University of Tabuk, Umluj, Saudi Arabia

**Keywords:** circular biorefinery, nano-bio interface, smart nanobiocatalysts, sustainable bioenergy, waste to biofuel conversion, waste valorization

## Abstract

Converting diverse waste streams, including agricultural residues, food waste, and industrial by-products, into biofuels is a major challenge for a circular bioeconomy. Smart green nanobiocatalysts are emerging as next-generation tools for sustainable waste-to-biofuel conversion. They combine enzyme specificity with the tunable properties of nanomaterials within circular biorefinery systems. Significant progress has been made in nano–bio interface engineering, AI-assisted catalyst discovery, and circular biorefinery frameworks. However, a comprehensive overview integrating these fields for rational biocatalyst design is still lacking. This review addresses this gap by providing a forward-looking synthesis of nano–bio interface engineering, stimuli-responsive catalytic systems, hybrid nanozyme–enzyme architectures, and AI-assisted catalyst design strategies to improve biochemical conversion and waste valorization. Emphasis is placed on structure–function relationships controlling enzyme immobilization, interfacial electron transfer, and multi-enzyme cascade organization. These features enhance catalytic efficiency, stability, and recyclability under industrial conditions. Applications include waste-derived lignocellulosic biomass, biodiesel feedstocks, food waste, wastewater streams, and anaerobic digestion. Additionally, techno-economic feasibility, life-cycle sustainability, carbon mitigation, and environmental safety are also evaluated. Overall, smart nanobiocatalysts provide a promising pathway toward efficient, climate-friendly, and digitally optimized biofuel production. They support sustainable waste management, resource recovery, and global net-zero energy goals, aligning biofuel innovation with circular bioeconomy objectives.

## Graphical Abstract

**Figure:**
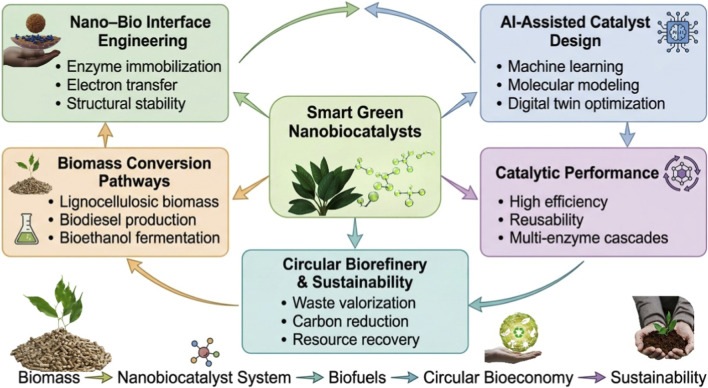

## 1 Introduction

The advancing global need for energy, together with rising greenhouse gas emissions and the increase of climate change have highlighted the need for the adoption of sustainable low-carbon and net zero energy systems at an accelerated pace (Aziz et al., 2024). Biofuels have been identified as a potential renewable alternative to fossil fuels due to their potential for carbon-neutral energy production and compatible with existing energy infrastructures (Cherwoo et al., 2023). However, current biofuel production processes are constrained by inefficient catalytic conversion, high energy consumption, and significant scale-up challenges, particularly in processing recalcitrant lignocellulosic biomass. (Torkashvand et al., 2022). Traditional catalytic systems, both chemical catalysts and free enzymes, often exhibit poor stability, reusability, enzyme denaturation under industrial conditions as well as poor substrate accessibility which limits the industrial viability and economic feasibility (Farhan et al., 2025; Khan, 2025).

In this context, green nanobiocatalysts have emerged as next-generation catalytic platforms that integrate the specificity of biological catalysts with the tunable physicochemical properties of nanomaterials. (Khafaga et al., 2024). These systems that are fabricated through environmentally benign routes using plant extracts, microbial systems or support derived from biomass offer increased catalytic efficiency, thermal and operational stability as well as recyclability (Jeevanandam et al., 2026). By allowing control over enzyme immobilization, surface interactions, and catalytic microenvironments, nanobiocatalysts have shown significant potential for improving biomass conversion, transesterification processes, and waste-to-biofuel routes; however, their performance is highly dependent on immobilization strategies, nanomaterial properties, feedstock composition, and process conditions. (Devi et al., 2021). However, challenges such as enzyme leaching, variability in catalytic performance across different nanomaterial systems, and limited long-term stability under industrial conditions remain significant barriers to large-scale implementation (Yadav et al., 2025). More recently, the idea of “smart” nanobiocatalysts has become a game-changing development offering adaptive and stimuli-responsive capabilities in combination with artificial intelligence (AI) powered design strategies. These smart systems are capable of dynamic reaction to environmental conditions such as, pH, temperature and substrate concentration, whereas AI and methods of machine learning are employable for rational design, optimization and prediction of nano-bio interactions, both on a molecular and process scale (Abdel-Mageed, 2025). Such integration enables the development of efficient and potentially self-optimizing catalytic systems adapted to complex and heterogeneous biofuel feedstocks. However, the field remains fragmented, with many studies addressing isolated aspects such as nanomaterial synthesis, enzyme immobilization, process optimization, or sustainability assessment, without a unified framework that connects molecular-level interactions with system-level performance and sustainability outcomes. (Villalba-Rodríguez et al., 2023).

Furthermore, the incorporation of nanobiocatalysts into circular bioeconomy models, in which waste streams are valorized into biofuels and value-added products, remains at an early stage of development, particularly regarding techno-economic feasibility, life-cycle sustainability, and climate-mitigation potential. The objective of this review is to provide an integrated and forward-looking synthesis of smart green nanobiocatalysts for sustainable waste-to-biofuel conversion. Specifically, this review aims to: (i) examine the fundamental principles and structure–function relationships governing nano–bio interface engineering; (ii) critically discuss stimuli-responsive, recyclable, hybrid nanozyme–enzyme, and multi-enzyme nanobiocatalytic systems; (iii) evaluate the role of AI-assisted design, molecular modeling, and digital twins in optimizing nanobiocatalyst performance; and (iv) assess the integration of smart nanobiocatalysts into circular biorefineries through techno-economic, life-cycle, environmental, and regulatory perspectives. The novelty of this review lies in its integrative framework that connects nano–bio interface engineering, AI-driven catalyst design, and circular biorefinery concepts within a unified systems-level perspective. Unlike previous studies focusing on isolated aspects, this review links molecular interactions with process-level implementation and sustainability, highlighting how smart nanobiocatalysts can enable efficient waste valorization and climate-resilient biofuel production.

## 2 Fundamentals of green nanobiocatalysts

### 2.1 Definition and classification

Green nanobiocatalysts represent hybrid catalytic systems in which biological catalysts and nanostructured materials are integrated through environmentally benign approaches to improve catalytic performance and operational stability. Based on their structural organization and mode of action, they are broadly divided into three categories (Beena Sreekumar et al., 2022). Enzymes-nanoparticles hybrid consists of the direct association of enzymes and nanomaterials, to obtain a better catalytic performance through better surface interactions and microenvironment control. Nanozymes formulated as inorganic or hybrid nanomaterials, simulating the function of natural enzymes, offer high stability, resistance to extreme conditions and low costs alternatives to ‘conventional’ enzymes (Hamza et al., 2022). Immobilized enzyme systems consist of enzymes fixed on nanostructured supports (magnetic nanoparticles, mesoporous silica, polymeric matrices etc.) and hence provide ease of enzyme recovery, low leaching, and better operational lifetime. These classifications provide a working framework for the design of efficient catalytic systems as processing solutions with respect to biofuel production processes (Silva et al., 2022). However, the performance of these nanobiocatalyst classes can vary significantly depending on enzyme–support compatibility, operational conditions, and long-term stability, which limits direct comparison across studies.

### 2.2 Green synthesis strategies

The sustainability of nanobiocatalysts is strongly influenced by the synthesis routes used for nanomaterial preparation. Green synthesis approaches are increasingly preferred because they reduce the use of toxic reagents, harsh reaction conditions, and energy-intensive processes (Kirubakaran et al., 2026). Plant mediated synthesis uses naturally occurring phytochemicals e.g., polyphenols and flavonoids as reducing and stabilizing agents to yield nanomaterials under mild conditions, hence reducing toxic by-products (Shahzadi et al., 2025). Microbial-one way to synthesize nanoparticles using bio-based approaches is microbial assisted synthesis, which utilizes the metabolic activity of microorganisms (bacteria, fungi, and algae) to biosynthesize nanoparticles with controlled morphology and size, and with a scalable and environmentally compatible approach (Ahir et al., 2025). Moreover, biomass-based nanomaterials encompassing biochar, cellulose nanofibers, and lignin-based supports have gained importance because of their renewability and biodegradability and their functionality. These materials, beyond the sustainable purpose of enzyme immobilization support, are also important for the valorization of waste in circular bioeconomy schemes (Jadoun et al., 2022). Despite these advantages, green synthesis approaches often face challenges related to reproducibility, scalability, and precise control over nanomaterial size, morphology, and surface chemistry, which may affect catalytic consistency.

### 2.3 Structure–function relationships

**FIGURE 1:**
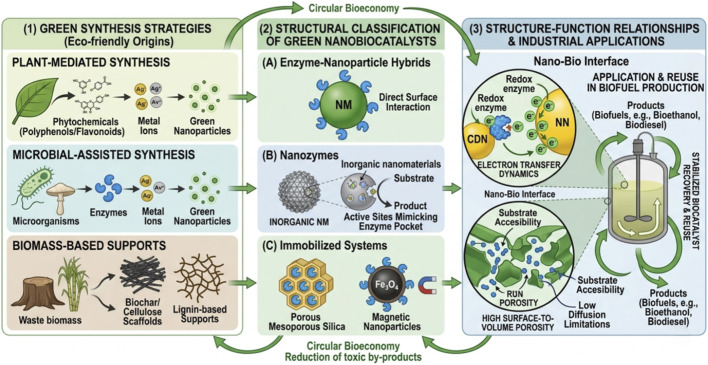
Schematic overview of circular-bioeconomy–driven green nanobiocatalysts. (1) Eco-friendly green synthesis strategies including plant-mediated synthesis, microbial-assisted synthesis, and biomass-based supports for producing green nanoparticles. (2) Structural classification of green nanobiocatalysts comprising enzyme–nanoparticle hybrids, nanozymes, and immobilized systems (e.g., porous silica and magnetic nanoparticles). (3) Structure–function relationships at the nano–bio interface highlighting enhanced electron transfer dynamics, improved substrate accessibility, reduced diffusion limitations, and high surface-to-volume porosity, leading to efficient catalytic performance and catalyst stabilization/reuse in biofuel production (e.g., bioethanol and biodiesel) with reduced toxic by-products. However, the actual catalytic performance may vary depending on nanomaterial properties, enzyme compatibility, and operational conditions.

The catalytic performance of green nanobiocatalysts is controlled by complex structures and functions of the nano-bio interface (Shahcheraghi et al., 2022). Surface chemistry is known to play a key role in determining enzyme binding, orientation and conformational stability and therefore have a direct impact on catalytic activity and resistance to denaturation. For example, surface functional groups and charge distribution influence enzyme orientation and binding strength, which directly affects catalytic turnover, stability, and substrate accessibility (Prabhakar et al., 2025). Porosity and exposing active sites play an essential part in controlling the substrate accessibility and mass-transference such behaviors; highly porous nanostructures comprise an increased surface area and decreased diffusion limitations (Zhao, 2025). Moreover, electron transfer dynamics are relevant in redox-mediated reactions during which conductive or semiconductive nanomaterials account for efficient electron mobility and thus an increase of catalytic turnover rates. A thorough knowledge of these parameters is crucial to the rational design of new high performance nanobiocatalysts able to cope with the complexities of the biofuel production systems (Zhao, 2025). However, the complexity of nano–bio interactions and variability in nanomaterial properties make it difficult to establish universal structure–function relationships, particularly under industrially relevant conditions. A schematic overview of circular-bioeconomy–driven green nanobiocatalysts, including synthesis strategies, structural classification, and nano–bio interface structure–function relationships, is shown in Figure 1 **.**

## 3 Nano-bio interfaces: mechanism governing catalytic performance

**FIGURE 2:**
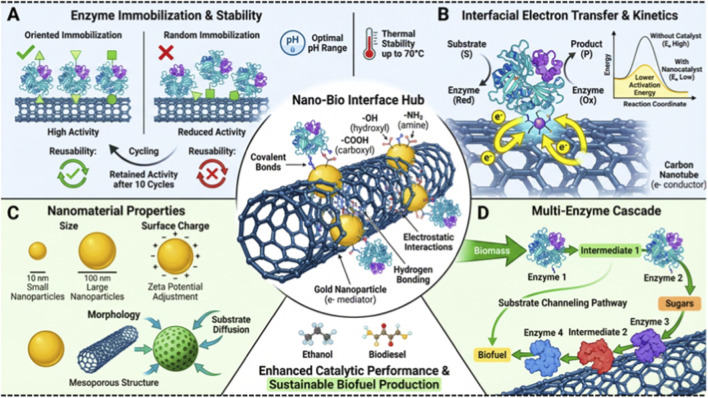
Schematic illustration of nano–bio interface–engineered immobilized enzyme systems for enhanced catalytic performance and sustainable biofuel production. **(A)** Comparison of oriented and random enzyme immobilization, highlighting improved activity, reusability, optimal pH tolerance, and thermal stability achieved through controlled attachment. **(B)** Interfacial electron transfer and reaction kinetics at conductive nanomaterial supports, where nanostructures facilitate electron flow, reduce activation energy, and enhance substrate-to-product conversion. **(C)** Influence of nanomaterial properties, including particle size, surface charge (zeta potential), morphology, and mesoporous structure, on enzyme loading and substrate diffusion. **(D)** Multi-enzyme cascade architecture enabling substrate channeling from biomass to intermediates and final biofuel products. The central nano–bio interface hub depicts covalent bonding, electrostatic interactions, and hydrogen bonding between enzymes and functionalized nanoparticles (e.g., gold nanoparticles on carbon nanotubes), collectively improving catalytic efficiency, stability, and recyclability for bioethanol and biodiesel production.

The catalytic efficiency and operational stability of the green nanobiocatalysts are controlled in principle by the interactions, which take place at the nano-bio interface through highly dynamic and functionally complex systems of biological macromolecules and nanostructured materials (Muteeb et al., 2026). Unlike the conventional immobilized enzyme platforms, the nanobiocatalyst occurrence mainly occurs by nanoscale interfacial mechanisms that control enzyme conformations, electron transfer, substrate accessibility and reaction kinetics (Muteeb et al., 2026). Such improvements are often observed under controlled laboratory conditions; however, their performance can decline under industrial stresses such as high temperature, extreme pH, and solvent exposure. Critically, inconsistent enzyme orientation in random immobilization can reduce activity due to active site blockage, while a mechanistic understanding remains essential for designing robust catalysts for biofuel production, where many systems fail beyond laboratory scale due to leaching or denaturation. (Damian et al., 2025). Figure 2 Illustrates nano–bio interface–engineered immobilized enzyme systems, showing how controlled immobilization, electron transfer, nanomaterial properties, and multi-enzyme cascades enhance catalytic performance and support sustainable biofuel production. These observations emphasize that catalytic performance is governed by the interplay between nanoscale design, interfacial interactions, and operating conditions.

### 3.1 Enzyme immobilization and conformational stability

Nanoscale immobilization stabilizes enzymes against biofuel process stresses, including temperature variations, pH fluctuations, and exposure to organic solvents. (Oke et al., 2023). The effectiveness of immobilization depends strongly on the method used; covalent bonding generally provides stronger attachment and improved reusability, whereas physical adsorption is more prone to enzyme desorption. (Wang et al., 2023). These immobilization strategies influence enzyme orientation, binding strength, and conformational flexibility, which directly determine catalytic turnover and substrate accessibility. Oriented immobilization improves substrate access by minimizing steric hindrance, whereas random immobilization may lead to partial blockage of active sites and reduced catalytic efficiency (Sheldon and van Pelt, 2013)).

Nanomaterials create a very favorable microenvironment that can preserve the native conformation of enzymes by decreasing structural denaturation and proteolytic degradation. The large surface area and the possibility to tune the functional groups of the nanostructured supporting allows for strong, yet flexible immobilization of enzymes which in turn increases the rigidity, while still retaining the flexibility of the catalyst (Fahim et al., 2025; Zhou et al., 2024). However, excessive enzyme loading or strong binding interactions may lead to structural rigidity or aggregation, limiting enzyme flexibility and reducing catalytic efficiency. Furthermore, challenges such as enzyme leaching, conformational distortion, and mass transfer limitations remain significant, particularly under prolonged or large-scale operation (Tadesse and Liu, 2025). These factors highlight the need for careful optimization of immobilization strategies in practical applications. (Li et al., 2024).

### 3.2 Interfacial electron transfer and catalytic kinetics

Electron transfer processes at the nano-bio interface are a critical determinant of catalytic efficiency in the case of redox mediated reactions of importance in the context of biofuel production. The nanomaterials, including nanoparticles of metals, carbon-based nanostructures, and conductive metal oxides, help in achieving high-speed electron transfer between the enzyme obtained active site and substrate thus increasing catalytic turnover rates (Xu et al., 2018). Electron transfer efficiency is strongly influenced by the spatial distance between enzyme active sites and conductive nanomaterials, where shorter distances promote faster electron transfer and improved catalytic rates. (Farhan et al., 2025).

These interfacial processes directly affect reaction kinetics, including substrate binding, intermediate formation, and product release. Improved electron mobility can enhance catalytic efficiency and reduce energy requirements in biofuel production systems (Narayanan and El-Sayed, 2003). However, in complex systems such as viscous biomass slurries, diffusion limitations and weak electrical coupling can significantly reduce electron transfer efficiency, making it a rate-limiting step under industrial conditions (Chang et al., 2026). These findings highlight the importance of optimizing nano–bio interface architecture to balance electron transfer efficiency with structural stability.

### 3.3 Role of Nanomaterial Physicochemical Properties.

The physicochemical properties of nanomaterials have an important central role in the functionality and efficiency of nano-bio interfaces. Parameters like particle size, surface charge, morphology, porous and surface functionalization have a great effect on the enzyme binding, stability and catalytic activity (Rahmati and Mozafari, 2019; Singh et al., 2024). Smaller nanoparticles provide higher surface area and enzyme loading capacity whereas controlled morphology and porosity are important for increased substrate diffusion and product transport. Surface functional groups such as hydroxyl group, carboxylic group and amino groups promote good enzyme support interactions and increase the enzyme immobilization efficiency (Gatoo et al., 2014).

The chemical composition and electronic properties of nanomaterials also affect catalytic performance, particularly through their role in electron transfer and structural stability. Carbon-based nanomaterials provide high conductivity, while metal oxide nanoparticles offer thermal robustness (Zhu et al., 2013). However, challenges such as nanoparticle aggregation, surface heterogeneity, and instability under reaction conditions can reduce effective surface area and limit catalytic performance. These limitations are particularly significant in heterogeneous biomass systems, where feedstock variability further complicates catalyst behavior.

### 3.4 Multi-enzyme cascade systems and spatial organization

One of the most important benefits associated with nano-bio interfaces is the fact that they may support multi-enzyme cascade systems, where several enzymes are co-immobilized on a unique nanostructured platform to undertake sequential biochemical reactions. Such systems follow nature’s metabolic pathways and can efficiently (reducing the loss of intermediate diffusion and enhancing efficiency of reactions) convert complex biomass into biofuels. Spatial organization of enzymes at the surface of nanomaterials enables us to control the proximity between catalytic sites, which improves substrate channeling, and prevents the built-up of inhibitory intermediates (Ge et al., 2025).

Nanostructured supports offer an ideal platform for the design of highly organized multi-enzyme systems because of their surface properties and the versatility of the support structure. By affecting the distribution, the orientation and the density of the enzymes, it is possible to optimize catalytic efficiency and to produce highly efficient biocatalytic pathways (Batool et al., 2023). These multi-enzymes nanobiocatalysts are of special interest in the conversion of biomass of lignocellulosic origin, in which several reactions must occur sequentially to convert the complex polymers into fermentable sugars. As such, the construction of spatially organized enzyme cascades is a fundamental step forward to the creation of intelligent and high performance nanobiocatalytic systems for sustainable biofuel production (Farhan et al., 2025). Overall, these nano–bio interface mechanisms demonstrate that catalytic performance is governed by a combination of structural design, interfacial interactions, and process conditions. Importantly, they provide the mechanistic foundation for AI-driven optimization and the development of scalable nanobiocatalytic systems within circular biorefinery frameworks.

## 4 Smart nanobiocatalysts: design, principle and functional innovations

The transition from conventional nanobiocatalysts to smart nanobiocatalysts is a model change towards dynamic, adaptive and highly efficient catalytic systems, which can react to complex and fluctuating process environments. In contrast with static catalytic platforms, smart nanobiocatalysts are designed with functional attributes that allow them to respond to external stimuli, to self-regulate and to increase their operational resilience (Ayub et al., 2023; Liu et al., 2025; Zhang and Tong, 2023). These systems operate by dynamically modulating enzyme conformation, substrate accessibility, and catalytic microenvironments in response to external triggers, thereby enabling real-time control of catalytic performance. As such, they integrate advanced material design, enzyme engineering, and emerging digital tools to optimize catalytic behavior in heterogeneous and multi-step biofuel production processes (Fahim et al., 2025; Zhang and Tong, 2023).

The design of smart nanobiocatalysts is driven by the principles of responsiveness, adaptability, recyclability, and functional integration thus guaranteeing efficiency of the catalytic process and long-term sustainability (Abdel-Mageed, 2025). However, the practical implementation of such systems remains constrained by challenges related to synthesis complexity, material stability, and reproducibility under industrial conditions.

**FIGURE 3:**
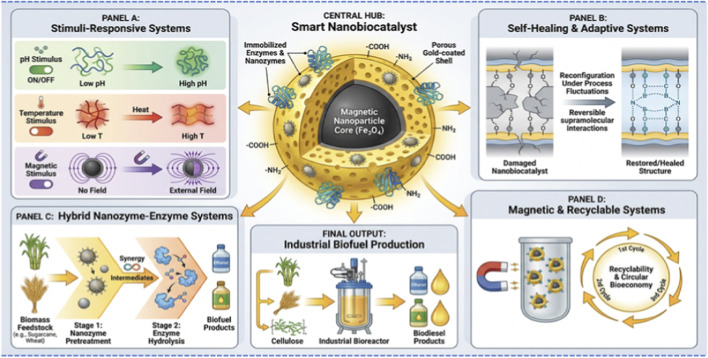
Schematic representation of a smart magnetic nanobiocatalyst platform with immobilized enzymes for sustainable biofuel production. The system integrates stimuli-responsive behavior (pH, temperature, magnetic field), self-healing adaptive structure, and hybrid nanozyme–enzyme cascade catalysis for efficient biomass conversion. Magnetic recovery and recyclability enable repeated use, supporting circular bioeconomy–driven industrial production of bioethanol and biodiesel.

A schematic representation of a smart magnetic nanobiocatalyst platform is shown in Figure 3, illustrating the integration of responsive materials with immobilized enzymes for sustainable biofuel production.

### 4.1 Stimuli-responsive nanobiocatalysts (pH, temperature, magnetic)

Stimuli-responsive nanobiocatalysts are designed so that their activity as catalysts can be dynamically changed in response to certain stimuli in the system, such as changing pH, temperature, and magnetic fields (Wu et al., 2023). These systems use smart materials such as pH-sensitive polymers, thermoresponsive hydrogels and magnetically active nanoparticles to control highly specific mechanisms of enzyme activity, substrate accessibility and catalytic rates. Mechanistically, these responses occur through changes in polymer swelling behavior, surface charge modulation, and conformational adjustments of immobilized enzymes, which directly influence catalytic kinetics and substrate binding (Karimi et al., 2016). For example, pH responsive systems can alter the conformation of the enzymes or surface charge under different pH conditions that can optimize the activity in different stages of biomass conversion (Hu et al., 2012).

Similarly, temperature-sensing nanomaterials can protect and control the enzyme activation or deactivation solution to improve thermal stability against denaturation for industrial purposes (Yang et al., 2025). Magnetically reactive nanobiocatalysts, frequently constructed due to iron oxide nanoparticles enable a control of the positioning and retrieval of the catalyst by utilizing magnetic areas. This capability eliminated not only the complicated issues of catalyst separation and re-use, but spatial control within the bioreactors, so that process efficiency may be improved (Bilal et al., 2019). The combination of such stimuli-responsive functionalities adds flexibility to a given process as well as the ability to precisely control the catalytic operations, making those systems extremely suitable to operate in complex biofuel production environments (Song et al., 2026). Despite these advantages, stimuli-responsive systems may suffer from limited long-term stability, sensitivity to extreme process fluctuations, and challenges in maintaining consistent responsiveness under large-scale operating conditions (Zhang et al., 2023). The integration of such functionalities enhances process flexibility and control; however, their performance is often highly dependent on precise tuning of environmental parameters, which can be difficult to maintain in industrial biofuel systems (Wang et al., 2024; Zhang and Tong, 2023).

### 4.2 Self-healing and adaptive catalytic systems

Self-healing and adaptive nanobiocatalysts are a part of a new frontier of the design of catalytic systems, where materials are designed with the ability to regain functionality after suffering structural or functional degradation. These systems include dynamic bonding systems, such as reversible covalent bonding or supramolecular systems, which make it possible to recover the catalyst in case of damage through mechanical stress, deactivation of the enzymes or change in the environment (Venkateswaran et al., 2024). Mechanistically, self-healing occurs through reversible bond reformation and molecular reassembly, allowing the catalyst to regain structural integrity and partially restore catalytic activity. In biofuel production systems, in which constant and durable productive performance is crucial, self-healing properties can provide the catalyst with a much longer lifespan, and consequently lower operation costs (Thomas, 2014). Adaptive catalytic systems have added function as the system continuously adapts their structural or chemical properties by adapting to process conditions. Such adjustment may mean adjustment of enzyme orientation, surface charge and catalytic microenvironment in order to preserve the optimal enzyme activity (Bordet and Leitner, 2023). This is an especially useful adaptability in processing the heterogeneous feedstocks, where there is a dynamic fluctuation of the composition of bottles and reaction conditions. The combination, therefore, of self-healing and adaptable properties also helps to create long-lasting nanobiocatalysts, able to perform at a high level with industrial conditions (Srivastav and More, 2025).

### 4.3 Hybrid nanozyme- enzyme systems

Hybrid nanozyme–enzyme systems combine the robustness of nanozymes with the high specificity of natural enzymes, creating multifunctional catalytic platforms (Sheng et al., 2024). In these systems, nanozymes provide stable and efficient catalytic activity under harsh conditions whereas enzymes provide high selectivity and specificity towards substrates. The support that arises between these components leads to higher catalytic efficiency, especially in multi-step reactions which require different catalytic actions (Zhang et al., 2025). Such hybrid approaches are especially beneficial in biofuel manufacturing processes that involve complex reaction pathways, the use of oxidative and hydrolytic reactions must be sequential or simultaneous, for example, in the destruction of lignocellulosic biomass (Nanda et al., 2014). Nanozymes can be used to catalyze the initial oxidation or pretreatment steps which increase accessibility of the substrate and facilitate the enzymes for hydrolysis or conversion reactions. This integration results in a decrease in process complexity while increasing reaction rates and improving the overall conversion efficiency, which could make hybrid nanozyme-enzyme systems an important innovation in nanobiocatalysis of the next-generation (Huang et al., 2019).

### 4.4 Magnetic and recyclable nanobiocatalysts

Magnetic and reusable nanobiocatalysts contain the design to overcome a major drawback against conventional catalytic systems, namely, poor recoverability and lack of reuse. By making the nanoparticles magnetic, which are usually made of iron oxides, such catalysts can be easily separated from the reaction mixtures by means of an external magnetic field, thereby eliminating costly separation methods such as centrifugation or filtration, which require a lot of energy (Mariño et al., 2021). This eliminates a large part of operational costs and improves the sustainability of the process.

In addition to facile recovery, these systems are designed to retain catalytic activity over multiple cycles through surface modifications and protective coatings that prevent enzyme leaching and structural degradation (Wang et al., 2020). Mechanistically, magnetic separation reduces catalyst loss while protective coatings stabilize enzyme conformation and prevent denaturation during repeated use. However, repeated magnetic recovery cycles may lead to gradual loss of catalytic activity due to particle aggregation, surface fouling, and structural degradation (Farhan et al., 2025; Sheldon and van Pelt, 2013). Furthermore, the long-term stability and cost-effectiveness of these systems remain key challenges for large-scale industrial applications. While their recyclability aligns with circular bioeconomy principles, their practical implementation requires further optimization to ensure consistent performance under real process conditions (Gama Cavalcante et al., 2024). To provide a comprehensive comparison of the smart nanobiocatalyst systems discussed across this section, a detailed summary is presented in Table 1, outlining their response mechanisms, catalytic advantages, limitations, and relevance to biofuel production.

**TABLE 1:** Summary of smart nanobiocatalyst systems, response mechanisms, advantages, limitations, and biofuel relevance.

Smart nanobiocatalyst system	Response/Design mechanism	Main catalytic advantage	Key limitation	Biofuel relevance	References
pH-responsive nanobiocatalysts	Surface charge modulation, polymer swelling/deswelling, and enzyme conformational changes under different pH conditions	Improved activity control across different reaction stages	Requires precise pH control; performance may decline under fluctuating waste-feedstock conditions	Biomass hydrolysis, fermentation support, and waste-to-biofuel conversion	Hu et al. (2012)
Temperature-responsive nanobiocatalysts	Thermoresponsive polymers or hydrogels regulate enzyme activation, flexibility, and stability	Improved thermal tolerance and controlled catalytic activity	Long-term stability may decrease under repeated heating/cooling cycles	Lignocellulosic biomass conversion and biodiesel production	Ingle et al. (2019), Singhvi and Kim (2020)
Magnetic nanobiocatalysts	External magnetic fields enable catalyst recovery, separation, and spatial control	Easy recovery, reuse, and reduced downstream separation cost	Aggregation, enzyme leaching, and possible activity loss after repeated cycles	Biodiesel production, enzyme recovery, and continuous bioprocessing	López et al. (2014), Rajkumari and Rokhum (2021)
Self-healing/adaptive systems	Reversible covalent bonds, supramolecular interactions, or dynamic carrier networks restore catalyst structure after damage	Extended catalyst lifetime and improved resilience under process stress	Complex synthesis and incomplete recovery after repeated damage cycles	Long-duration biofuel production and heterogeneous feedstock processing	Misson et al. (2015), Mustapha et al. (2025)
Hybrid nanozyme–enzyme systems	Nanozymes provide robust catalytic activity, while enzymes provide substrate specificity	Enables sequential or complementary catalytic reactions	Possible incompatibility between nanozyme and enzyme activity; selectivity control remains difficult	Lignocellulosic pretreatment, oxidative conversion, and cascade biofuel pathways	Dutta et al. (2023), Huang et al. (2019)
Multi-enzyme smart cascades	Spatial organization of multiple enzymes promotes substrate channeling and sequential conversion	Reduced intermediate loss and improved pathway efficiency	Enzyme incompatibility and different optimal operating conditions	Bioethanol production from lignocellulose and integrated waste valorization	Xu et al. (2020)

To summarize the functional differences among smart nanobiocatalyst systems discussed in Section 4, Table 1 provides a concise comparison of their response mechanisms, catalytic advantages, limitations, and relevance to biofuel production.

## 5 AI-driven design and digitalization of nanobiocatalysts

**FIGURE 4:**
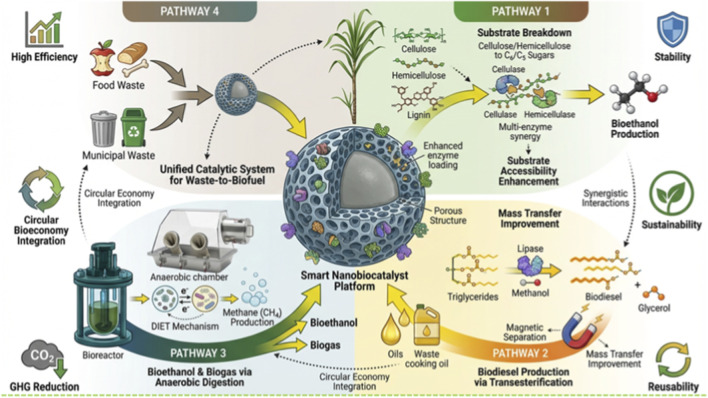
Overview of a smart nanobiocatalyst platform for converting food and municipal wastes into biofuels. The system integrates lignocellulosic bioethanol production, biodiesel synthesis from waste oils, and biogas generation via anaerobic digestion. Improved enzyme loading and mass transfer enhance efficiency, supporting sustainability and circular bioeconomy integration.

The use of artificial intelligence (AI) and digital technologies in the development of nanobiocatalysts shifts the approach to nanobiocatalyst development from the empirical, trial-and-error method to the predictive, data-driven method of design (Chen and Tang, 2024). Given the complexity of nano-bio interfaces and the multivariate nature of biofuel production systems, the conventional experimental strategies are not always feasible to optimize catalytic performance in an efficient way (Bosu et al., 2022). AI driven methodologies that help to fast screen materials, predict the enzyme-nanoparticle interactions and optimize the catalytic conditions, thus greatly accelerate the development of high performance nanobiocatalysts (Abdel-Mageed, 2025). Furthermore, digitalization facilitates the integration of molecular-level insights with process-scale modeling, supporting the development of intelligent and autonomous biorefinery systems aligned with sustainability objectives (Butean et al., 2025). However, the application of AI in nanobiocatalysis remains constrained by limited availability of high-quality datasets, challenges in model generalization across different nanomaterial systems, and the complexity of accurately capturing biological variability. An overview of a smart nanobiocatalyst platform integrating these processes is shown in Figure 4.

### 5.1 Machine learning for catalyst optimization

Machine learning (ML) algorithms have become a powerful tool for optimization of nanobiocatalyst design, finding complex relationship between material properties, enzyme and catalytic performance (Tadesse and Liu, 2025). Supervised learning models, such as regression and classification algorithms can be used to predict catalytic activity, stability and reusability from input features such as nanoparticle size, surface functionalization from enzyme loading and reaction conditions. Unsupervised learning approaches offer the further possibility to discover hidden patterns and clustering of high performing catalyst configurations (Erdem Günay and Yıldırım, 2021). Mechanistically, ML models function by mapping nonlinear relationships between structural descriptors (e.g., surface charge, porosity, functional groups) and catalytic outputs, allowing identification of key parameters governing enzyme immobilization efficiency and reaction kinetics (Chai et al., 2021; Sampaio and Fernandes, 2023). ML reduces experimental workload by narrowing the design space and prioritizing candidate systems for validation. In biofuel production, these models are used to optimize enzyme immobilization efficiency, substrate conversion rates, and reaction conditions for processes such as lignocellulosic biomass degradation and biodiesel production. Additionally, advanced techniques such as deep learning and reinforcement learning are being explored to develop adaptive catalytic systems capable of real-time optimization under dynamic process conditions (Paton et al., 2025; Ralbovsky and Smith, 2023)Despite these advantages, ML approaches may suffer from overfitting, limited interpretability, and reduced transferability across different catalytic systems, which can restrict their practical applicability (Sampaio and Fernandes, 2023).

### 5.2 Molecular modeling of nano–bio interactions

Molecular modelling and simulation techniques have given crucial information on the underlying interactions that control nano-bio interfaces and are allowing rational design of nanobiocatalysts at the atomic and molecular levels (Wang et al., 2019). Methods like molecular docking, molecular dynamics (MD) simulations and quantum mechanical calculations can be used for a detailed study of enzyme adsorption, conformational changes and binding affinities on the surface of nanomaterials (Bhattacharjee et al., 2023). These approaches mechanistically explain how surface chemistry, charge distribution, and nanomaterial morphology influence enzyme orientation, conformational stability, and catalytic activity, thereby directly linking structural properties to functional performance (Sousa et al., 2017).

By simulating electron transfer pathways and reaction mechanisms, it is also conceivable that molecular modelling can be used to predict catalytic efficiency and find possible bottlenecks in the kinetics of a reaction (Khan and Khan, 2025; Lakhani et al., 2024). Such insights are of special value in the design of nanomaterials with optimized surface properties, which will promote increased stability and activity of enzymes. When combined with experimental data, such computational tools define a good framework for understanding and engineering nano-bio interfaces to a high level of precision (Mohan et al., 2024). However, computational predictions may not always fully capture complex real-world conditions, particularly in heterogeneous biomass systems, where multiscale interactions and process variability introduce additional uncertainties (Jain et al., 2024; Khafaga et al., 2024).

### 5.3 Data-driven enzyme engineering

AI-driven approaches increasingly find application for enzyme engineering which allows the design of enzymes with improved stability, activity and specificity for nanobiocatalytic systems. Data driven methods make use of large data sets containing information on protein sequences, protein structures and protein functional properties to predict useful mutations and to inform protein engineering efforts (Khan and Khan, 2025). Techniques like sequence based deep learning models and structure guided algorithms allow to identify the modifications at the amino acid level that result in an improvement of the enzymes performance in industrial settings (Wang et al., 2023).

In nanobiocatalysis, enzyme engineering efforts can be focused on the improvement of the mimicry to nanomaterial supports, resistance against denaturation and optimization of the catalytic efficiency in the heterogeneous environment. The coupling between AI processing and directed evolution coupled with high throughput screening further speeds up the development of robust enzymes which can work effectively in smart nanobiocatalytic systems themselves. This convergence of biotechnology and artificial intelligence is a significant step forward in this development of next-generation biofuel catalysts (Ayub et al., 2023; Hosseini et al., 2026). Nevertheless, challenges such as limited experimental validation, data bias, and the complexity of protein–material interactions may limit the reliability of predictive models.

### 5.4 Digital twins for biorefinery systems

Digital twin technology, which entails the development of virtual replicas of physical systems, is becoming a powerful tool for optimization of nanobiocatalyst performance at the process and system level. In applications where biorefinery is used, digital twins combine real time data from sensors, process models and artificial intelligence algorithms to simulate and predict system behavior based on several different operating conditions. This allows dynamic optimization of catalytic processes such as conversion of feedstocks, enzyme activities, and yields of products (Hosseini et al., 2026; Singh et al., 2025).

By using a connection between molecular and process-scale catalyst design and simulations, digital twins enable a holistic approach to Catalyst design and the management of sustainable biofuel production systems. They enable scenario analysis, predictive maintenance and real time decision-making, thus contributing to a more efficient process with a reduced level of operational risks (Pasha et al., 2021). Furthermore, digital twins can be coupled with models of life cycle assessment and techno and economic models to assess the sustainability and economic viability of nanobiocatalytic systems to support their implementation on a large scale in circular biorefineries.

From an industrial perspective, the application of AI-driven nanobiocatalysts remains largely at the laboratory and pilot scale, with limited transition to commercial biorefineries (Mustapha et al., 2025; Pramanik et al., 2023). Key barriers include the lack of standardized and high-quality datasets, challenges in integrating AI models with existing process control systems, and the complexity of real-time monitoring of nano–bio interfaces (Sampaio and Fernandes, 2023). Furthermore, variability in feedstock composition and process conditions in industrial biofuel production introduces additional uncertainties that reduce the reliability of predictive models (Williams et al., 2016). Addressing these challenges will require the development of robust data infrastructures, hybrid experimental–computational frameworks, and validation of AI-assisted designs under realistic process conditions.

## 6 Applications for sustainable biofuel production

**FIGURE 5:**
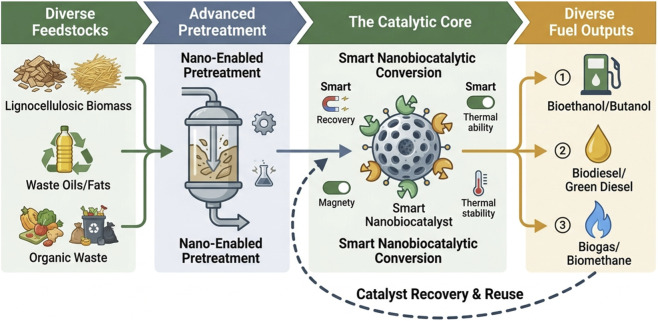
Schematic representation of a nano-enabled bioconversion platform for waste valorization into biofuels. Diverse feedstocks (lignocellulosic biomass, waste oils/fats, and organic waste) undergo advanced pretreatment followed by smart nanobiocatalytic conversion with enhanced thermal stability and magnetic recoverability. The system enables efficient production of bioethanol/butanol, biodiesel/green diesel, and biogas/biomethane with catalyst recovery and reuse.

Advanced green nanobiocatalysts have shown concepts for change in a variety of biofuel production routes through improved catalytic efficiency, process integration and use of complex and heterogeneous feedstocks. Their distinctive ability to integrate high specificity, stability and tunability functionality allows key bottlenecks that are inherent to conventional catalytic systems to be resolved (Muteeb et al., 2026). From the conversion of lignocellulosic biomass to advanced waste-to-biofuels approaches, nanobiocatalysts are being developed more for better yield, less energy input and sustainable and circular bioenergy systems (Gokul et al., 2026). However, most reported systems remain at laboratory or pilot scale (TRL 2–4), with limited translation to industrial biorefineries due to challenges in catalyst stability, cost, and process integration. As illustrated in Figure 5, nano-enabled bioconversion platforms integrate diverse feedstocks and catalytic processes to produce bioethanol, biodiesel, and biogas.

### 6.1 Lignocellulosic biomass conversion

Lignocellulosic biomass, which is made of cellulose, hemicellulose and lignin, is one of the most abundant and renewable feedstocks to produce biofuels (Jatoi et al., 2023). However, its structural recalcitrance and heterogeneity significantly limit efficient conversion.

Nanobiocatalysts enhance enzymatic hydrolysis by improving enzyme–substrate interactions and reducing diffusion barriers. Mechanistically, nanostructured supports promote controlled enzyme orientation and facilitate substrate accessibility, leading to improved hydrolysis rates under optimized conditions (Sankaran et al., 2021). Cellulases and hemicellulases immobilized on nanomaterials exhibit improved resistance to thermal and chemical stress. However, enzyme deactivation, leaching, and reduced activity over extended cycles remain significant challenges, particularly under industrial conditions involving high temperature, inhibitors, and complex feedstock matrices (Sankaran et al., 2021; Singhvi and Kim, 2020). Furthermore, variability in biomass composition (e.g., lignin content and crystallinity) introduces inconsistencies in catalytic performance, limiting process predictability and scalability. Multi-enzyme nanobiocatalytic systems enable synergistic degradation of biomass into fermentable sugars, supporting downstream bioethanol production (Guo et al., 2024; Zoghlami and Paës, 2019).

### 6.2 Biodiesel production

Biodiesel production via transesterification of triglycerides is a key application of nanobiocatalysts, particularly lipase-based systems. Compared to conventional chemical catalysts, enzymatic nanobiocatalysts operate under milder conditions and reduce unwanted by-products (Meher et al., 2006).

Nanostructured supports improve enzyme dispersion and enhance interaction with hydrophobic substrates. This reduces mass transfer limitations in biphasic systems by improving interfacial contact between reactants and catalytic sites (Christopher et al., 2014; Mahdi et al., 2023; Xie and Ma, 2009). Magnetic nanobiocatalysts enable catalyst recovery and reuse. However, repeated recycling can lead to nanoparticle aggregation, enzyme deactivation, and gradual loss of catalytic efficiency. Additionally, high costs associated with enzyme immobilization and nanomaterial synthesis remain key barriers for large-scale biodiesel production (Christopher et al., 2014; Mahdi et al., 2023). Compared to conventional catalysts, nanobiocatalysts offer higher selectivity but may exhibit lower long-term stability under industrial processing conditions (Damian et al., 2025; Farouk et al., 2024).

### 6.3 Bioethanol and biogas systems

Nanobiocatalysts are important to enhance the performance of bioethanol and biogas production systems through the improvement of the enzymatic and microbial processes. In the production of bioethanol nanobiocatalysts help in hydrolysis of biomass to fermentable sugars and enhance the rate of fermentation by stabilizing the enzymes and supporting microbe activity (Rai et al., 2019). Integration of nanomaterials can also improve mass transfer and reduce the inhibitory effect and thus result in increased ethanol yields. In the case of biogas systems, it is especially the case with anaerobic digestion where nanobiocatalysts play a role in better microbial metabolism and process stability. Nanomaterials can serve as electron conduits for achieving direct inter-species electron transfer (DIET) between microbial communities, which will increase the rate of methane production. In addition, enzyme-based nanobiocatalysts catalyze the degradation of complex organic matter which improves substrate utilization and overall process efficiency (Li et al., 2025). These advancements contribute to stable and productive development of bioenergy systems that have lower operational constraints.

### 6.4 Waste-to-biofuel conversion

The use of smart nanobiocatalysts in the conversion of wastes to biofuels is a turning point in the development of circular bioeconomy and sustainable resource management. Unlike traditional processes requiring purified or homogeneous feedstocks, the use of nanobiocatalysts can be very efficient in processing heterogeneous and low value waste streams converting them into valuable biofuels (Ramesh et al., 2026).

Food waste with high carbohydrates, lipids, and proteins contents can be successfully transformed into bioethanol, biodiesel, and biogas with the assistance of nanobiocatalytic systems. Enzyme immobilization on nanomaterials primarily improves hydrolysis of the substrate and improves conversion efficiency, even in case of complex and variable waste matrices (Joshi and Arora, 2023). Similarly, agricultural residues such as straw and husks of crops and lignocellulosic residues, i.e., by-products, can be efficiently treated with the help of nanobiocatalysts for efficient deconstruction of biomass and sugar release (Singh et al., 2025).

Municipal organic waste such as waste from the kitchen and biodegradable fractions of solid waste are great challenges to be faced because of their heterogeneity and contamination. Smart nanobiocatalysts, and those possessing adaptive and multi-functional characteristics, thus allow for robust catalytic performance under such variable catalytic conditions (Muhammad et al., 2024). They achieve the enhancement of the enzymatic degradation and the increased activity of micro-organism in the process of anaerobic digestion and the efficient conversion into biofuels such as methane or bioethanol (Dharshini et al., 2023).

However, the performance of nanobiocatalysts in waste-to-biofuel systems is often affected by feedstock variability, catalyst fouling, and potential inhibition due to contaminants, which may limit long-term operational stability and process scalability (Mahdi et al., 2023). Importantly, the integration of nanobiocatalysts into waste-to-biofuel systems does not only improve process efficiency, but it also plays an important role in waste valorization, relief of the urban waste landfill burden and reduction of greenhouse gas emissions. This approach starts to be in close agreement with circular bioeconomy principles, whereby waste streams will be turned into renewable energy resources, closing the loop between resource consumption and energy production. Table 2 summarizes the comparative performance, economic feasibility, and sustainability metrics of conventional catalysts versus smart nanobiocatalysts in diverse biofuel production pathways, highlighting key advantages, limitations, and potential for circular biorefinery integration.”

**TABLE 2:** Comparative Performance, Economic Feasibility, and Sustainability Metrics of Conventional vs. Smart Nanobiocatalysts in Biofuel Production.

Catalyst type	Feedstock	Conversion efficiency (%)	Reusability (cycles)	Cost ($/kg or relative)	Carbon footprint (kg CO_2_-eq/kg fuel)	Advantages	Limitations	References
Free Enzymes	Lignocellulosic biomass	40–60	1–2	Low ($10–20/kg)	Moderate	High specificity, easy availability	Rapid denaturation, low stability, poor recyclability	Kumar et al. (2008)
Chemical Catalysts (acid/base)	Vegetable oils/Biomass	70–85	1	Low to moderate	High	Fast reaction rates, mature technology	Harsh conditions, by-products, difficult separation, low feedstock versatility	Thangadurai and Tye (2021)
Immobilized Enzymes (Conventional)	Lignocellulose, oils	60–75	5–10	Moderate ($20–50/kg)	Moderate	Improved stability, some reusability	Limited adaptive response, moderate cost, slower kinetics than chemical catalysts	Hossain et al. (2025)
Nanozyme-based Catalysts	Lignocellulose, food/agri waste	65–80	10–15	Moderate ($30–60/kg)	Low	Enhanced stability, faster electron transfer, tolerates harsh conditions	Limited substrate specificity, early-stage industrial data	Guo et al. (2024)
Hybrid Nanozyme–Enzyme Systems	Lignocellulose, waste oils	75–90	15–20	High ($50–100/kg)	Low	Multi-step cascade reactions, high efficiency, adaptive	Higher synthesis complexity, initial cost, scale-up challenges	Zhu et al. (2026)
Stimuli-responsive Smart Nanobiocatalysts	Lignocellulose, agricultural residues, municipal waste	80–95	20–30	High ($60–120/kg)	Very Low	Self-healing, pH/temperature/magnetic control, recyclable, high substrate versatility	High synthesis complexity, regulatory and safety considerations, limited long-term industrial validation	Ayub et al. (2023), Nazarzadeh Zare et al. (2021)
Magnetic and Recyclable Nanobiocatalysts	Waste oils, food and municipal waste	85–92	20–25	High ($50–100/kg)	Very Low	Easy recovery, high reusability, compatible with circular biorefinery	Moderate enzyme leaching possible, upfront cost, requires magnetic infrastructure	Abd Elkodous et al. (2021)
AI-Optimized Multi-Enzyme Smart Nanobiocatalysts	Lignocellulose, mixed waste feedstocks	90–98	25–30	Very High ($70–150/kg)	Very Low	Maximized conversion efficiency, autonomous adaptation, high resilience, circular integration	Data-driven design required, scale-up remains challenging, early commercial readiness	Long et al. (2025), Periyasamy et al. (2022)

## 7 Circular biorefineries and sustainability integration

**FIGURE 6:**
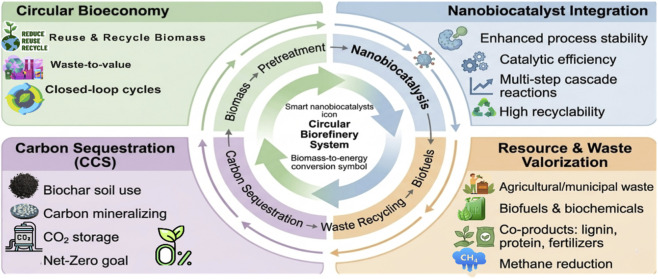
Integrated framework of a smart nanobiocatalytic system for sustainable biomass valorization. Low-value feedstocks undergo advanced pretreatment and conversion via adaptive nanobiocatalysts to produce multiple value-added products (biofuels, biochemicals, and fertilizers). The system incorporates renewable energy use, waste heat recovery, catalyst reuse, and carbon capture, supporting process efficiency, carbon neutrality, and circular bioeconomy goals.

The step from conventional biofuels production to circular biorefinery is a critical step towards what we can call sustainable energy systems, where resource economy, wellness valorization and mitigation of climate changes build perfect synergies for one system. Circular biorefineries operate according to the principles of circular bioeconomy approach and convert biomass and wastes into a variety of value-added products, including biofuels, biochemicals and fertilisers, and limit their environmental impact. Within this paradigm smart nanobiocatalysts are revealed as enabling technologies to enhance the process efficiency, promote the multi product valorization and the integration of carbon-neutral and low-carbon energy strategies (Mohan et al., 2016; Vural Gursel et al., 2022). As shown in Figure 6, circular biorefineries integrate advanced pretreatment, nanobiocatalytic conversion, and sustainability-driven processes to enable efficient biomass valorization.

### 7.1 Concept of circular bioeconomy

The use of circular bioeconomy addresses the circular economy concept, focused on reusing, recycling and valorizing biological resources for the purpose of sustainable economic growth with lower environmental pressures. Unlike to linear production systems, circular biorefineries are predestined to close material and energy loops and, this way, convert residual biomass, agricultural by or organic waste streams to bioelectricity and high value biochemicals (Maina et al., 2017; Toplicean and Datcu, 2024). This approach means that the reliance on fossil fuel usage is reduced, less waste is created and gases significant greenhouse gases are reduced and this contributes to paying towards the net zero for the country and the world. Circular bioeconomy framework is heavily dependent on deployment of technology innovation in terms of high mathematics of catalysis, advanced process designing as well as renewable energy inputs in order to optimize the efficiency of resources and ensure environmental sustainability (Davis et al., 2018; Elegbeleye et al., 2025).

### 7.2 Integration of nanobiocatalysts into biorefineries

Smart nanobiocatalysts are a promising strategies in the possibilities for improving the efficiency and flexibility of circular biorefinery. By offering high catalytic activity, robustness in heterogeneous condition as well as adaptive functionality, nanobiocatalysts promote the conversion of a variety of feedstocks such as lignocellulosic biomass, agricultural residues, food waste into biofuels and value-added products (Damian et al., 2025). Their incorporation into the processes employed in biorefinery allows for cascade reactions and multi-step conversions on a single platform, hence lowering energy input, reaction time and the complexity of the process.

In addition, the immobilization of the enzymes on the nanomaterials allows for their repeated use and constant processing, which enhances their economic feasibility to a greater extent. Integration of magnetic or stimuli-responsive nanobiocatalysts to further improve separation efficiency and recyclability allowing operational design in line with circularity principles (Shakeel et al., 2025). By combining molecular level catalysis and system-level process engineering, nanobiocatalysts help integrate different bioconversion pathways at a molecular level seamlessly and sustainably, which can support the operation of whole-cell biorefinery processes (Muteeb et al., 2026). However, integration of nanobiocatalysts into biorefineries remains limited by process complexity, catalyst stability under continuous operation, and techno-economic constraints at industrial scale.

### 7.3 Resource recovery and waste valorization

One of the fundamental principles of circular biorefineries is that resource recovery of all process streams is maximized. Nanobiocatalysts play a role in that by catalyzing the efficient degradation and conversion of residual biomass, waste oils, and organic fractions from waste (from municipalities or agriculture) into biofuels or biochemical intermediates (Romero et al., 2022; Solarte-Toro et al., 2023). Their high specificity and catalytic stability under heterogeneous conditions represents an important means of utilization of feedstocks of low value that would otherwise be thrown away.

Beyond the production of biofuels, the use of nanobiocatalysts to produce co-products like lignin-derived chemicals, protein hydrolysates or nutrient-rich residues suitable for soil amendment. This approach not only makes the whole biorefinery process much more economical for the overall economic value but also helps mitigate the environmental burdens by minimizing the landfill deposition, preventing the emissions of methane and the recycling of nutrients (Liu et al., 2025; Woźniak et al., 2025). The ability of smart nanobiocatalysts to function in multi-step systems or modular reaction systems is especially beneficial for complicated feedstock because it allows for co-production of energy and bioproducts in accordance with circular economy goals.

### 7.4 Coupling with carbon capture and sequestration

The incorporation of nanobiocatalysts in circular biorefineries can also be used to improve carbon capture and sequestration (CCS) initiatives in favor of climate-aligned energy strategies (Idris et al., 2025). Advanced catalytic systems optimize the utilization of carbon in the biofuel synthesis process and allow better conversion of biomass carbon into liquid or gaseous fuels. In addition, by facilitating the production of biochar and other carbon-rich co-products, nanobiocatalysts help with long-term carbon sequestration when the uses of these materials are returned to soils or used in construction and industrial applications (Marin et al., 2025).

Coupling biorefinery activities with CCS technologies i.e., biochar amendment or carbon mineralization ensures closed-loop system, where CO_2_ emissions are minimized and carbon is actively captured in renewable energy production cycles (Ayaz et al., 2025). This synergy is aligned with sustainability targets and net zero strategies, whereby it can clearly be seen as being of paramount importance of using smart nanobiocatalysts not only to better the efficiency of biofuels, but also to mitigate the impact of climate change within the framework of circular bioeconomy.

## 8 Techno-economic and life cycle assessment

A proper assessment of the performance of smart nanobiocatalysts for sustainable biofuel production requires a complete techno-economic analysis (TEA) and life-cycle analysis (LCA) to quantify their cost-effectiveness, energy efficiency, environmental impact, and the comparison of their performance to that of conventional catalytic systems. The connection of TEA and LCA provides a comprehensive overview of the feasibility of both economic and sustainability criteria of nanobiocatalytic platforms and ensures that technological innovations are converted into workable and climate aligned solutions.

### 8.1 Cost analysis of nanobiocatalysts

While nanobiocatalysts can perform better and maintain longer stability, the production and immobilization costs are still a major factor to consider for industrial use. Cost factors are related to the synthesis of raw nanomaterials, production and immobilization of enzymes, surface functionalization, and recovery and recycling (Fahim et al., 2025). Green synthesis approaches, for example, through the mediation of plants or biomass during the production of nanoparticles, can have significant direct consequences by reducing the cost of materials and energy consumption and limiting the number of hazardous by-products (Nasrollahzadeh et al., 2019). Magnetic and recyclable nanobiocatalysts can further enhance cost efficiency through various cycles of reuse so that less catalyst is needed. Techno-economical studies have shown that optimized multi-enzymes and hybrid systems of nanozymes and enzymes despite being more expensive in the beginning could be cost-competitive in biofuel production per-unit costs because of increase in conversion rates, lower enzyme loading and operational stability (Arnodo et al., 2023; Sharma et al., 2022). Process modelling and sensitivity analysis would thus be crucial to identify economically viable configurations and operational parameters to be implemented at a large scale. However, economic feasibility remains highly dependent on catalyst lifetime, enzyme recovery efficiency, nanomaterial cost, and the number of reuse cycles achieved under realistic process conditions.

### 8.2 Energy efficiency and carbon footprint

The nanobiocatalysts increase energy efficiency for biofuels production through reducing the energy activation, rates of reaction and allowing for a mild operational condition over conventional chemical catalysts (Goyal et al., 2025). Reduced energy input is a direct translation to a reduced emission of greenhouse gases and an increase in the sustainability of the process. Additionally, high catalytic turnover and reusability minimize the intensity (energy and material wise) of repeatedly producing catalysts (Mariño et al., 2021). Life-cycles assessment studies have shown that biofuel systems based on smart nanobiocatalysts have a greatly lower carbon footprint in the cradle-to-gate phase and cradle-to-grave phase (Gholami and Pourfayaz, 2024). For example, the production of lignocellulosic biomass hydrolysis processes and biodiesel transesterification processes catalyzed by immobilized or hybrid nanobiocatalysts have lower CO_2_ equivalent emission processes due to decreased chemical inputs, increased yields and process integration in circular biorefineries (Rai et al., 2019). Regardless, reported carbon-footprint reductions are strongly influenced by system boundaries, nanomaterial synthesis routes, energy inputs, and assumptions used in LCA models.

### 8.3 Environmental trade-offs

Despite the obvious benefits, the use of nanomaterials creates possible environmental trade-offs that should be carefully considered. Nanoparticle synthesis - especially when using metal-based catalysts - may involve toxic reagents, high consumption of energy and release of the residue of nanomaterials into the environment (Nasrollahzadeh et al., 2019). Green synthesis routes and biodegradable supports can address these risks, though thorough evaluations of the environmental fate and ecotoxicity are important (Samuel et al., 2022). Furthermore, life cycle related impacts associated with water consumption, waste production, and end-of-life for nanomaterials should be evaluated to ensure that the overall sustainability is met. Multicriteria in Life cycle assessment (LCA) enables the quantification of such trade-offs between improved catalytic performance and potential environmental burdens and informs the responsible designs and feature deployments of nanomaterials for biocatalysis (i.e., nano-biocatalysis) (Dabare et al., 2025; Hischier and Walser, 2012). Therefore, future LCA studies should include nanoparticle recovery, end-of-life fate, ecotoxicity, and potential secondary pollution to avoid overestimating sustainability benefits.

### 8.4 Comparison with conventional systems

When benchmarked against conventional biofuel catalysis (e.g., free enzyme systems, chemical acid/base catalysts, or bulk heterogeneous catalysts), nanobiocatalysts can demonstrate improved performance under optimized conditions in terms of reaction efficiency, stability, recyclability, and feedstock versatility (Bhan et al., 2025). They can be used to produce yields in milder conditions and to take the more complicated and waste-derived substrates which are difficult for traditional catalysts. From the sustainability point of view, smart nanobiocatalysts as part of circular biorefineries may reduce resource consumption, hazardous by-products, and greenhouse gas emissions depending on process conditions and system design during a life cycle when compared to conventional systems. Although upfront costs are likely to be higher, these benefits may offset higher upfront costs under favorable process and operational conditions, especially for industrial scale biofuel production in the context of net zero and circular economy goals (Coronado-Contreras et al., 2025; Maturi et al., 2026). However, these advantages are often influenced by catalyst lifetime, recovery efficiency, and scale-up feasibility, which remain key challenges for industrial implementation.

## 9 Environmental, health and regulatory considerations

Smart nanobiocatalysts possess significant potential in the sustainable production of biofuels; however, their widespread implementation requires careful consideration of the environmental, health and regulation issues. Nanotoxicity and ecological risksare among the main issues given that nanoparticles can interact with the soil, water and microbial ecosystems and can break natural processes or bioaccumulate in food chains (Abbas et al., 2020). The environmental fate of nanomaterials such as degradation, leaching and persistence, require full monitoring of nanomaterials to ensure the safe integration in biorefinery operations. Existing regulatory frameworks belonging to nanomaterials continue to be scattered with minimal standards consistent to production, use, and disposal of nanomaterials; in essence, there are policy gaps, hindering big scale commercialization (Chávez-Hernández et al., 2024). In view of these challenges, the idea of safe-by-design nanobiocatalysts has emerged in which emphasis is laid on the use of biodegradable, low toxicity materials, on green synthesis routes and in controlling thesurface functionalities to limit the adverse impacts. Incorporation of these principles ensures that nanobiocatalysts have a high performance without compromising its environmental safety and public health standards. However, the lack of globally harmonized regulatory frameworks and long-term environmental risk assessments remains a key barrier to industrial adoption. TThis approach supports the alignment of technological innovation with sustainability goals and circular bioeconomy principles (Prajapati et al., 2025).

## 10 Current challenges and critical research gap

Though great advances, the practical use of smart nanobiocatalysts for sustainable production of biofuels is facing several crucial challenges. Scaling-up remains one of the major barriers, as efficiencies achieved at the laboratory scale often decline under industrial conditions due to mass transfer limitations, mixing inefficiencies, and reactor design constraints (Arya et al., 2021; Damian et al., 2025). The discipline also has issues with the lack of standardization in synthesis techniques, analytical protocols, as well as performance measures, which makes it difficult to reproduce and compare results between investigations. Enzyme stability and leaching still remain in limiting the long-term operational efficiency by multi-cycles and harsh processing environment (Bartoli et al., 2023). Furthermore, real-world applications of these systems remain limited, as most studies are conducted under controlled laboratory conditions. As a result, the robustness of these systems across diverse feedstocks remains uncertain. In addition, limited availability of high-quality experimental data constrains AI-driven design and predictive modeling, reducing the reliability and generalizability of machine learning and digital twin approaches (Kreuzer et al., 2024; Wang et al., 2025).

From a technological readiness perspective, most smart nanobiocatalyst systems for biofuel production remain at laboratory or early pilot-scale development (Misson et al., 2015; Rijuta Ganesh Saratale et al., 2022), with limited evidence of continuous operation under industrial biorefinery conditions (Mahdi et al., 2023). Among the currently studied systems, magnetic and immobilized enzyme-based nanobiocatalysts show relatively higher practical potential because of their recoverability and reuse, whereas stimuli-responsive, self-healing, hybrid nanozyme–enzyme, and AI-optimized systems remain largely at proof-of-concept stages. Key barriers to industrial adoption include high nanomaterial and enzyme immobilization costs, enzyme leaching, catalyst fouling, long-term stability loss, feedstock variability, reactor compatibility, regulatory uncertainty, and limited validation under real process conditions (Makepa and Chihobo, 2024; Saratale et al., 2022). Therefore, future studies should prioritize pilot-scale testing, standardized performance metrics, catalyst lifetime assessment, and techno-economic validation before commercial deployment. Addressing these deficiencies is imperative towards the pursuit of smart nanobiocatalysts for the scalable and economically viable and sustainable production platforms for biofuels from proof-of-concept investigations.

## 11 Future perspectives: towards next-generation smart biofuel systems

The future of sustainable biofuel production is in the development of next-generation smart biofuel systems providing a perfect union of advanced nanobiocatalysis with AI processes optimization and principles of circular bioeconomy. Autonomous biorefinery “AI integrated autonomous biorefineries have the promise to provide real-time monitoring, prediction control coupled with adaptive optimization of the catalytic processes that enable high efficiency under variable feedstock and environmental conditions.” Design of multifunctional nanobiocatalysts that perform cascade reactions as well as self-healing and stimulus-responsive adaptation will allow for further increasing the process flexibility and for a wider variety of substrates. Integration with synthetic biology offers the opportunity to create a microbial consortia and enzymatic set-up for tailor made feedstock usage and reaction pathway engineering in order to obtain synergy nano-bio system for maximum production yield of biofuels. These such innovations in total support the climate aligned production systems of biofuels, reduced carbon footprint, carbon sequestration and low emission energy support. By developing forward looking efficient, circular and resilient biofuel platforms, smart nanobiocatalysts will play a starring role in meeting Sustainable Development Goals (SDGs) and net zeros, in turn bridging the technological innovation and global sustainability and energy security achievements.

## 12 Conclusion

Smart green nanobiocatalysts represent an emerging approach in sustainable biofuel production, linking nanotechnology, enzymology, and circular bioeconomy concepts. Their integration overcomes critical limitations inherent in the function of conventional catalysis in terms of low stability, poor substrate specificity and low feedstock versatility while enabling efficient conversion of lignocellulosic biomass, biodiesel feedstocks, bioethanol feedstocks and a variety of organic wastes. Taking advantage of the precision of enzymes and the unique physicochemical properties of nanomaterials, these systems have improved catalytic performance and operational stabilityand the capacity to carry out multistep and cascade reaction. Stimuli-responsive, self-healing, and hybrid nanozyme–enzyme systems also introduce adaptive functionality under dynamic conditions to assure robust functionality in dynamic industrial conditions. AI-driven design and digitalization enable predictive modeling, optimization of nano–bio interfaces, and potential real-time process control through digital twins. This coming together of molecular knowledge and intelligence on the system level enables autonomous, efficient and highly adaptable biorefineries. Moreover, the use of nanobiocatalysts embedded in circular biorefinery frameworks allows us to implement comprehensive waste valorization, resource recovery and carbon management frameworks to align the production of biofuels with net zero targets and Sustainable Development Goals. Despite these advances, several challenges remain, including scale-up limitations, enzyme leaching, feedstock variability, lack of standardization, and limited data availability for AI models. Addressing these gaps using safe-by-design principles along with sound techno-economic strategies and environmentally responsible deployment strategies will be crucial in relation to commercialization. In conclusion, smart nanobiocatalysts present a route towards future generation systems for sustainable and climate-aligned biofuels combining technological innovation and ecological sustainability and circularity and can play a decisive role in making bioenergy a cornerstone of future low carbon, resilient and resource efficiently functioning energy systems.
